# Vitamin D Predicts All-Cause and Cardiac Mortality in Females with Suspected Acute Coronary Syndrome: A Comparison with Brain Natriuretic Peptide and High-Sensitivity C-Reactive Protein

**DOI:** 10.1155/2013/398034

**Published:** 2013-11-17

**Authors:** Patrycja A. Naesgaard, Ricardo A. León de la Fuente, Stein Tore Nilsen, Leik Woie, Torbjoern Aarsland, Harry Staines, Dennis W. T. Nilsen

**Affiliations:** ^1^Department of Cardiology, Stavanger University Hospital, 4068 Stavanger, Norway; ^2^Institute of Medicine, University of Bergen, 5021 Bergen, Norway; ^3^Cardiology Research Institute, Catholic University of Salta, A4400ANG Salta, Argentina; ^4^Department of Research, Stavanger University Hospital, 4068 Stavanger, Norway; ^5^Sigma Statistical Services, Balmullo KY16 0BJ, UK

## Abstract

Vitamin D may not only reflect disease but may also serve as a prognostic indicator. Our aim was to assess the gender-specific utility of vitamin D measured as 25-hydroxy-vitamin D [25(OH)D] to predict all-cause and cardiac death in patients with suspected acute coronary syndrome (ACS) and to compare its prognostic utility to brain natriuretic peptide (BNP) and high-sensitivity C-reactive protein (hsCRP). Blood samples were harvested on admission in 982 patients. Forty percent were women (65.9 ± 12.6 years). Mortality was evaluated in quartiles of 25(OH)D, BNP, and hsCRP, respectively, during a 5-year follow-up, applying univariate and multivariate analyses. One hundred and seventy-three patients died; 78 were women. In 92 patients (37 women), death was defined as cardiac. In women, the univariate hazard ratio (HR) for total death of 25(OH)D in Quartile (Q) 2 versus Q1, Q3 versus Q1, and Q4 versus Q1 was 0.55 (95% CI 0.33–0.93), 0.29 (95% CI 0.15–0.55), and 0.13 (95% CI 0.06–0.32), respectively. In females, it was an independent predictor of total and cardiac death, whereas BNP and hsCRP were less gender-specific. No gender differences in 25(OH)D were noted in a reference material. Accordingly, vitamin D independently predicts mortality in females with suspected ACS.

## 1. Introduction

Cardiovascular disease (CVD) is the primary cause of mortality both in men and women worldwide [1]. Women have been underrepresented in clinical trials [2]. They very often present with atypical symptoms and are frequently undiagnosed. Furthermore, their risk factors are understudied. Due to women's higher age of disease manifestation, they are also more likely to have other diseases and comorbidities, rendering the diagnosis more difficult and complex. This has also resulted in less research related to the predictive utility of the established biomarkers in this gender.

Recently, numerous observational and epidemiological studies suggest that vitamin D deficiency may be related to CVD and mortality [3–9] and is associated with myocardial infarction (MI) [10, 11] and sudden cardiac death (SCD) [12]. As vitamin D deficiency in men and women is increasing, we believe that the general diet does not contain a sufficient amount of this vitamin and/or that people do not spend enough time outdoors to create a sufficient amount of this vitamin upon sun exposure. In a previous study with 2-year follow-up [13], we found low levels of 25-hydroxy-vitamin D [25(OH)D] in a chest-pain population from Northern Argentina. This was an unexpected finding, as we assumed that our study population would be protected from vitamin D deficiency due to high sun exposure (latitude 24 degrees south of the Equator, altitude >1000 meters above sea level).

Vitamin D may add prognostic information in CVD patients beyond that of the traditional biomarkers consisting of the troponins, brain natriuretic peptide (BNP), and high-sensitivity CRP (hsCRP).

The troponins are sensitive biomarkers of myocardial injury in the acute coronary syndrome (ACS). BNP is released into the circulation in response to ventricular dilatation and pressure overload [14, 15] and is a well-known marker of left ventricular dysfunction and heart failure (HF) [16–19] as well as a prognostic marker in the ACS [20]. C-reactive protein is an acute-phase reactant and a marker of underlying systemic inflammation, including atherosclerosis and plaque rupture [21–23]. HsCRP assays have been shown to be of prognostic value in CVD and ACS [20, 24].

The objective of this study was to assess the gender-specific utility of vitamin D, assessed by 25(OH)D (where D represents D_2_ and D_3_) to predict all-cause mortality and cardiac death as well as SCD during a 5-year follow-up of a chest-pain population with suspected ACS from Northern Argentina, employing univariate and multivariate analyses, and to evaluate its prognostic utility as compared to BNP and hsCRP. In addition, levels of 25(OH)D in our patient populations were compared with the 25(OH)D levels collected from healthy blood donors.

## 2. Methodology

### 2.1. Study Design and Patient Population

The details of the ARgentinean Risk Assessment Registry in the Acute Coronary Syndrome, the “ARRA-RACS Study” (Ref. Clinical Trial.gov identifier: NCT01377402), have been published previously [13, 20]. Briefly, the study was performed at nine hospitals in the Province of Salta, Northern Argentina. It was designed to assess the prognostic utility of 25(OH)D, BNP, and hsCRP levels in 982 consecutive patients, hospitalized with chest pain and suspected ACS, from December 2005 to January 2009. Furthermore, we collected blood samples from 104 blood donors (51% females), drawn from the same locality.

The primary outcome was a 5-year all-cause mortality from the time of inclusion, and the secondary outcomes included cardiac death and SCD. The total patient population was divided into two subgroups, females and males, respectively.

Main exclusion criteria were age <18 years, unwillingness or incapacity to provide informed consent, and prior inclusion in the present study. Baseline characteristics of the total population have previously been described [13].

The study was approved by the Ethics Committee at the Board of Medical School of Salta and by other local institutions [13] and was conducted in accordance with the Helsinki Declaration of 1971, as revised in 1983. The Norwegian biobank containing Argentinean blood samples was approved by the Regional Board of Research Ethics and by the Norwegian health authorities. This study was monitored by Stavanger Health Research, Stavanger, Norway. Written informed consent was obtained from all patients.

### 2.2. Blood Samples and Laboratory Measurements

Blood samples were drawn immediately following admission by direct venipuncture of an antecubital vein, applying a minimum of stasis. A second blood sample for repeated troponin T (TnT) determination was drawn six hours following the first sample. Baseline laboratory data included measurements of 25(OH)D_2_, 25(OH)D_3_, TnT, hsCRP, glucose and lipids in serum, BNP measured in EDTA (ethylenediamine tetraacetic acid) plasma, and estimated glomerular filtration rate (eGFR), calculated by the Modification in Diet in Renal Disease (MDRD) formula.

TnT was quantified by a cardiac-specific second-generation troponin T ELISA assay from Roche Diagnostics, using a high-affinity cardiac-specific TnT isoform antibody [25]. The lower detection limit of the assay used is 0.01 ng/mL. In this study, a cut-off level of 0.05 ng/mL was used with a coefficient of variation of 10% for the diagnosis of an MI, whereas patient groups in this study were defined according to TnT release (TnT > 0.01 ng/mL).

BNP (Microparticle Enzyme Immunoassay Abbott AxSYM, Abbott Laboratories, Abbott Park, IL, USA) and hsCRP (Tina-quant C-reactive protein (latex) high sensitive assay, Roche Diagnostics, Germany) were analyzed as recommended by the manufacturer and as previously described [20].

25(OH)D analysis was carried out at the Department of Medical Biochemistry at the Stavanger University Hospital. Assessment of 25(OH)D status was performed by the determination of the metabolites 25(OH)D_3_ and 25(OH)D_2_ in serum, applying liquid-liquid extraction, derivatization with 4-phenyl-1,2,4-triazoline-3,5-dione reagent (PTAD, Sigma-Aldrich, St. Louis, MO, USA), followed by liquid chromatography coupled with tandem mass spectrometry detection, as previously described [13].

25(OH)D levels were also analyzed in blood samples collected from blood donors.

### 2.3. Statistical Analysis

Patients were divided into quartiles according to their 25(OH)D, BNP, and hsCRP levels, respectively. Approximately normally distributed variables were given as mean and standard deviation (SD), while variables with more skewed distributions were given as median and quartiles. The Chi-square test for association was applied between the 25(OH)D, BNP, and hsCRP quartiles, respectively, and categorical variables at baseline. The one way ANOVA was used to test for the equality of means of scale variables (e.g., age) amongst the quartiles and the two-sample *t*-test and Mann-Whitney tests were used for comparing the means and medians, respectively, of two samples. The hazard ratios (HR) are presented with 95% confidence interval (CI). Stepwise Cox multivariable proportional hazards regression models with total death, cardiac death, and SCD, as the dependent variable, and 25(OH)D, BNP, hsCRP, and other variables as potential independent predictors (listed below) were fitted. To examine the differences in prognosis between subjects in the upper quartiles versus the lowest quartile of 25(OH)D, BNP, and hsCRP, respectively, we adjusted for gender, age, smoking, hypertension, index diagnosis, diabetes mellitus (DM), congestive heart failure (CHF) (defined by Killip-Kimball class [26] at admission; patients in classes 2 to 4 were classified as CHF patients and patients in class 1 were classified as non-CHF), history of previous coronary heart disease (CHD) (i.e., history of either angina pectoris, MI, coronary artery bypass graft (CABG), or percutaneous coronary intervention (PCI)), hypercholesterolemia/use of statins, TnT > 0.01 ng/mL, eGFR, hsCRP, BNP, body mass index (BMI) (kg/m^2^), month of sampling, and use of beta-blockers prior to enrolment. The Kaplan-Meier product limits were used for plotting time-to-event and the logrank test was used to compare survival curves across quartiles. The statistical analyses were performed using the statistical package SPSS version 19.0. All tests were two-sided with a significance level of 5%. Highly significant differences were based on a significance level of 1%.

## 3. Results

A total of 982 patients (588 men and 394 women) were enrolled in the ARRA-RACS study. Four samples were not available and two patients were lost to follow-up. At index hospitalization, 388 patients (39.6%) had a peak TnT exceeding 0.01 ng/mL. Of these, 258 (43.9%) were males and 130 (33.1%) were females.

Mean age of the total population was 62.2 ± 13.4 years. Mean age for females and males was 65.9 ± 12.7 years and 59.7 ± 13.3 years, respectively. The median 25(OH)D concentration in the total population was 50.7 (38.3–62.4) nM (25 and 75 percentiles), differing in females and males (Mann-Whitney test; *P* < 0.001); 44.2 (34.0–57.5) nM (25 and 75 percentiles) versus 54.3 (43.0–64.4) nM (25 and 75 percentiles). The median BNP concentration in females and in males was 81.0 (36.7–199.9) pg/mL (25 and 75 percentiles) and 74.8 (35.1–168.4) pg/mL (25 and 75 percentiles), respectively, and the median hsCRP concentration was 3.5 (1.6–8.3) mg/L (25 and 75 percentiles) and 3.0 (1.3–8.8) mg/L (25 and 75 percentiles), respectively.

The distribution of clinical baseline characteristics in the quartiles of 25(OH)D, BNP, and hsCRP, respectively, for the total population has previously been described [13, 20]. 

Baseline characteristics stratified according to 25(OH)D quartiles at admission in females and males are shown in Tables 1 and 2, respectively.

After a follow-up period of 5 years, 173 patients (17.6%) had died, 78 were women and 95 were men. In 92 patients (9.4%) (37 women and 55 men), death was defined as cardiac, of whom 59 patients (6.0%) (24 women and 35 men) were characterized as SCD.

### 3.1. Female Patient Population

In the univariate analysis (Table 3), there was a statistically significant difference in HR when comparing each of the upper three quartiles to the lowest quartile (Q1) of 25(OH)D with respect to all-cause mortality, cardiac death, and SCD. The HR for total death of 25(OH)D in Q2 versus Q1, Q3 versus Q1, and Q4 versus Q1 was 0.55 (95% CI, 0.33–0.93), *P* = 0.027, 0.29 (95% CI, 0.15–0.55), *P* = 0.000, and 0.13 (95% CI, 0.06–0.32), *P* = 0.000, respectively. Comparing the highest quartile (Q4) to Q1 of BNP, the HR for all-cause mortality, cardiac death, and SCD was also statistically significant. A similar result was obtained for all-cause mortality when comparing the respective quartiles of hsCRP, whereas this biomarker was not found to predict cardiac death.

In the multivariate analysis (Table 3), the HR for all-cause mortality and cardiac death in Q4 compared to Q1 of 25(OH)D was 0.16 (95% CI, 0.06–0.42), *P* = 0.000, and 0.08 (95% CI, 0.01–0.59), *P* = 0.014, respectively, whereas 25(OH)D status did not add any prognostic information related to SCD. With regard to BNP, HR for all-cause mortality in Q4 compared to Q1 was 4.58 (95% CI, 1.56–13.45), *P* = 0.006, whereas hsCRP yielded no prognostic information.

**Figure 1:**
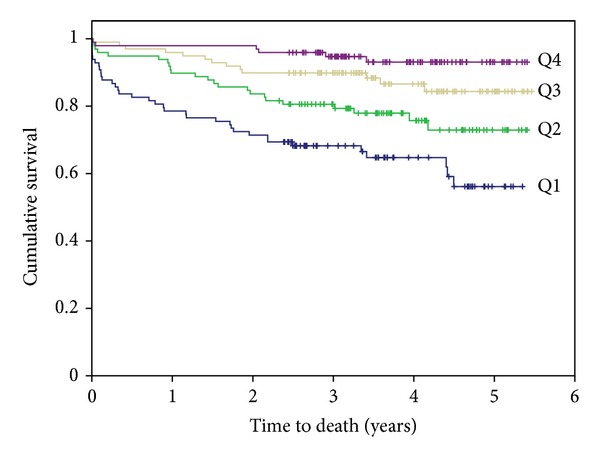
Kaplan-Meier plots for the cumulative risk of all-cause mortality in women in the 25(OH)D quartiles.

**Figure 2:**
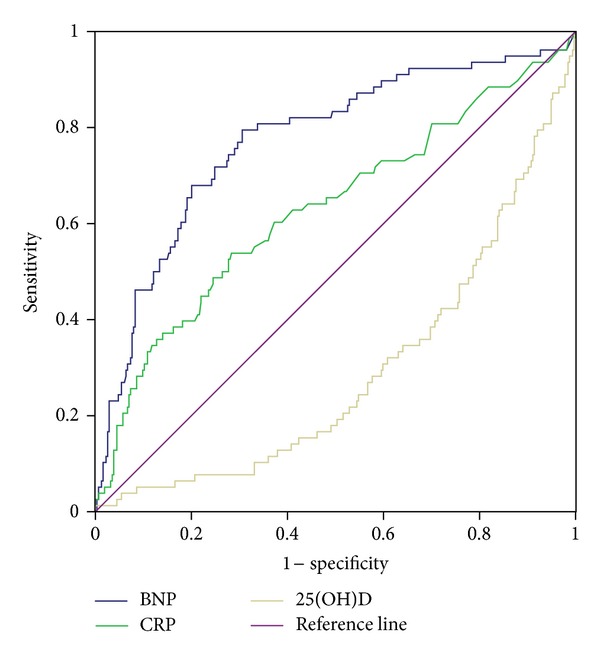
Receiver operating characteristic curves for 25(OH)D, BNP, and hsCRP for evaluation of all-cause mortality in the female patient population.

The Kaplan-Meier survival plots for the cumulative risk of all-cause mortality in women in the 25(OH)D quartiles are presented in Figure 1 (logrank test *χ* ^2^ (3) = 36.217; *P* < 0.001). The receiver operating characteristics curves (ROCs) for all-cause mortality related to 25(OH)D, BNP and hsCRP quartiles, respectively, are presented in Figure 2. The areas under the ROC for 25(OH)D, BNP, and hsCRP were 0.291 (*P* = 0.000), 0.773 (*P* = 0.000), and 0.630 (*P* = 0.001), respectively.

### 3.2. Male Patient Population

In the univariate quartile analysis (Table 3), results obtained in males, comparing Q4 to Q1, showed the same pattern as in females, whereas Q2-Q3 differed only slightly compared to Q1.

 In the multivariable analysis (Table 3), 25(OH)D status did not add any prognostic information in males. The HR of BNP for cardiac death and SCD in Q4 compared to Q1 was 4.22 (95% CI, 1.42–12.58), *P* = 0.010, and 4.55 (95% CI, 1.01–20.44), *P* = 0.048, respectively. The HR of hsCRP for all-cause mortality in Q4 compared to Q1 was 2.10 (95% CI, 1.12–3.94), *P* = 0.020.

**Figure 3:**
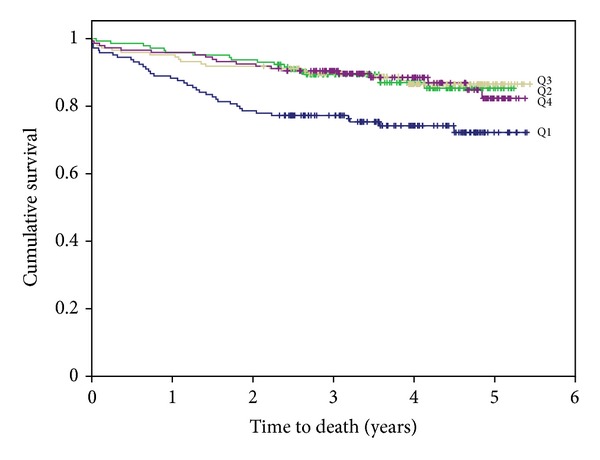
Kaplan-Meier plots for the cumulative risk of all-cause mortality in men in the 25(OH)D quartiles.

**Figure 4:**
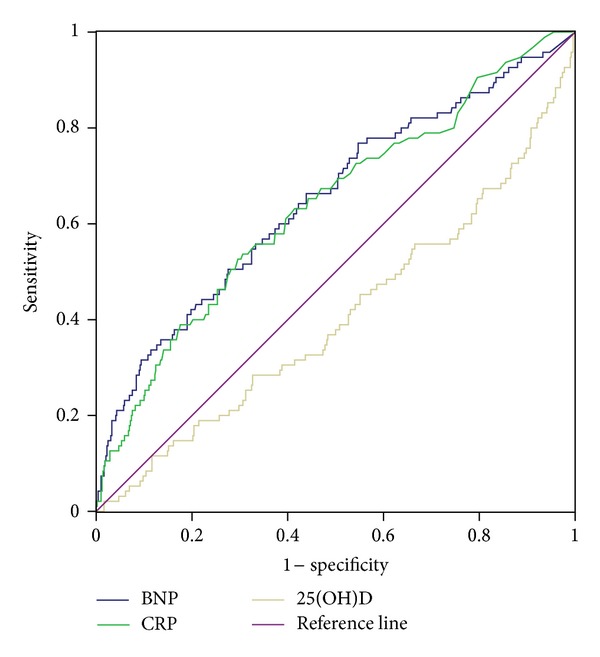
Receiver operating characteristic curves for 25(OH)D, BNP, and hsCRP for the evaluation of all-cause mortality in the male patient population.

The Kaplan-Meier survival plots for the cumulative risk of total mortality in men in the 25(OH)D quartiles are presented in Figure 3 (logrank test *χ* ^2^ (3) = 15.704; *P* < 0.001). The ROC curves for all-cause mortality related to 25(OH)D, BNP, and hsCRP quartiles, respectively, are presented in Figure 4. The areas under the ROC for 25(OH)D, BNP, and hsCRP were 0.405 (*P* = 0.003), 0.650 (*P* = 0.000), and 0.638 (*P* = 0.000), respectively.

### 3.3. Blood Donors

Mean age of the total blood donors group (*n* = 104) was 46.1 ± 10.1 years. There were 53 females with a mean age of 46.7 ± 9.7 years, BMI of 27.2 ± 4.2 kg/m^2^, and a 25(OH)D level of 54.5 ± 19.3 nM. The mean age of the male population (*n* = 51) was 45.4 ± 10.4 years, and this gender presented with a BMI of 28.7 ± 4.1 kg/m^2^ and a 25(OH)D level of 52.5 ± 14.9 nM. No gender differences in 25(OH)D levels were found in this normal population.

## 4. Discussion

In this prospective observational study from Northern Argentina, in which we address the prognostic utility of 25(OH)D, BNP, and hsCRP during a 5-year follow-up in patients presenting with chest pain and suspected ACS, we found that 25(OH)D was independently related to the risk of death in females and that this risk marker may be of less importance in males. BNP and hsCRP behaved as general risk markers with a slightly different pattern in females and males.

In a 2-year survival analysis of the same patient material, 25(OH)D deficiency and elevated BNP and hsCRP levels were associated with reduced survival, both for all-cause and for cardiac mortality [20] in the total population, even after correcting for confounders including cardiovascular risk factors, medication, age, gender, BMI, and sampling time [13].

Females represented 40% of the patient population in the present study. They were older and had lower 25(OH)D levels than men. The baseline 25(OH)D quartile characteristics for men and women were similar, except for hypertension and hsCRP. Significantly more male patients had hypertension and higher levels of hsCRP in the lowest quartile of 25(OH)D. In the BNP and hsCRP quartiles, there were several differences in the baseline characteristics (not shown). Males with high BNP presented with more risk factors for cardiovascular disease. A similar relationship was found in the hsCRP quartiles for both genders, especially in women. These data are in accordance with the current review literature stating that women present with more multifaceted disease [27, 28].

25(OH)D was found to be an independent predictor for total and cardiac death in women. BNP was found to be independently related to all-cause mortality in women and to cardiac and sudden cardiac death in males.

The gender differences in prognostic utility in relation to 25(OH)D and BNP were not quite as consistent for hsCRP. This biomarker was found to be an independent predictor of all-cause mortality limited to the male population.

In our univariate analysis of the prognostic utility of 25(OH)D in women, there was a gradual and statistically significant increase in predictive utility by a factor of 2 from the first to second quartile and from the second to third quartile, respectively, whereas, from the third to fourth quartile, the total mortality increased by 1.5. This relationship was not noted in men, in whom the difference was similar between the first and each of the three upper quartiles.

Our multivariate analysis has demonstrated an abundance of confounding factors related to 25(OH)D in males, diluting the utility of 25(OH)D as an independent prognostic indicator in this gender.

The gradual increase in death through the quartiles in women and the maintenance of predictive utility as judged by our multivariate analysis could be explained by their lower intraquartile values of 25(OH)D compared to males. The lower 25(OH)D concentration in women could be due to its binding to vitamin D receptors in fatty tissue which is more predominant in the female gender. Another explanation may be related to the domestic/indoor occupation of women with less exposure to sun and the use of skin protection such as face cream and make-up. In addition, women in this study were five years older than men in the corresponding 25(OH)D quartiles, and, with a reduction of 7-dehydrocholesterol in the epidermis and dermis due to age, skin of older individuals does not convert 25(OH)D as effectively as skin in younger people [29]. However, as there were no gender differences in 25(OH)D levels in the normal blood donor population, in which age and BMI were matched, we have to consider the possibility that 25(OH)D levels may be associated with the burden of disease.

In the ROC analysis, we demonstrated that 25(OH)D values are associated with a higher mortality in females (0.291) compared to men (0.405) (low AUC scores in the ROC analysis reflect a higher mortality in the lowest compared to the highest quartile, and the opposite relationship is found when the risk is higher in the upper compared to the lowest quartile).

The main strength of this study is the high proportion of females (40%), with an evaluation of outcome in both genders. Moreover, the mortality rate was high, allowing us to include several covariates in our multivariate model. Furthermore, only two patients were lost to follow-up.

Limitations include only one blood sample at admission and levels of 25(OH)D, BNP, and hsCRP before and after the acute event remain unknown. Although we did not adjust for left ventricular ejection fraction, we did adjust for known CHF and CVD as well as for BNP and hsCRP for the evaluation of the prognostic utility of 25(OH)D. Furthermore, we did not measure parathyroid hormone.

## 5. Conclusion

Vitamin D was found to be an independent predictor of all-cause and cardiac mortality in females but not in males. Lower values of vitamin D in women in the main study may reflect a greater health deficiency, as no gender difference was found in normal subjects.

## Figures and Tables

**Table 1:** Baseline characteristics of female patients arranged according to quartiles of 25(OH)D.

Characteristics *n* (%)	Quartiles of 25(OH)D				*P* value
	Q1	Q2	Q3	Q4	
25(OH)D (nM)*	27.1 ± 5.3	39.0 ± 2.7	50.7 ± 3.6	68.2 ± 8.4	0.000
Age, years*	70.5 ± 11.3	67.8 ± 12.6	64.8 ± 11.9	60.3 ± 12.7	0.000
Smoking status, *n* (%)					0.328
Current smoker, *n* (%)	8 (8.3)	11 (11.3)	15 (15.5)	13 (13.7)	
Past smoker, *n* (%)	79 (82.3)	69 (71.1)	73 (75.3)	72 (75.8)	
Never smoked, *n* (%)	9 (9.4)	17 (17.5)	9 (9.3)	10 (10.5)	
Angina pectoris, *n* (%)	16 (16.3)	21 (21.4)	23 (23.2)	20 (20.4)	0.669
CHF, *n* (%)					
Killip class 2–4	24 (24.5)	21 (21.4)	9 (9.1)	17 (17.3)	0.031
History of previous MI, *n* (%)	10 (10.2)	6 (6.1)	4 (4.0)	4 (4.1)	0.230
CABG, *n* (%)	4 (4.1)	4 (4.1)	2 (2.0)	0 (0.0)	0.207
PCI, *n* (%)	7 (7.1)	5 (5.1)	9 (9.1)	7 (7.1)	0.757
Hypertension, *n* (%)	71 (72.4)	71 (72.4)	74 (74.7)	61 (62.2)	0.219
History of DM 1, *n* (%)	2 (2.1)	1 (1.0)	1 (1.0)	0 (0.0)	0.566
History of DM 2, *n* (%)	31 (32.0)	15 (15.5)	15 (15.2)	7 (7.2)	0.000
STEMI, *n* (%)	11 (11.3)	8 (8.5)	12 (12.4)	9 (9.3)	0.804
TnT release, *n* (%)	45 (45.9)	33 (33.7)	24 (24.5)	28 (28.6)	0.009
eGFR (*μ*mol L^−1^)*	74.6 ± 32.7	78.9 ± 30.9	83.3 ± 30.3	77.8 ± 26.2	0.240
Cholesterol^†^/statin, n (%)	18 (18.4)	17 (17.3)	13 (13.1)	19 (19.4)	0.664
Beta-blocker, *n* (%)	21 (21.6)	26 (26.8)	28 (28.3)	25 (26.0)	0.742
Known CHD, *n* (%)	22 (22.7)	30 (30.6)	29 (29.3)	25 (25.8)	0.594
BMI (kg/m^2^)*	28.3 ± 5.2	27.6 ± 5.8	27.9 ± 5.2	27.3 ± 4.1	0.641
BNP quartiles					0.004
Q1	19 (19.4)	16 (16.3)	31 (31.3)	32 (32.7)	
Q2	17 (17.3)	25 (25.5)	27 (27.3)	30 (30.6)	
Q3	27 (27.6)	27 (27.6)	23 (23.2)	21 (21.4)	
Q4	35 (35.7)	30 (30.6)	18 (18.2)	15 (15.3)	
hsCRP quartiles					0.062
Q1	21 (21.6)	21 (21.4)	29 (29.3)	25 (25.5)	
Q2	21 (21.6)	23 (23.5)	28 (28.3)	27 (27.6)	
Q3	18 (18.6)	32 (32.7)	21 (21.2)	27 (27.6)	
Q4	37 (38.1)	22 (22.4)	21 (21.2)	19 (19.4)	

*Mean ± SD.

^†^Concentration > 250 mg/dL.

SD: standard deviation; 25(OH)D: 25-hydroxyvitamin D; CHF: congestive heart failure; MI: myocardial infarction; CABG: coronary artery bypass grafting; PCI: percutaneous coronary intervention; DM: diabetes mellitus; STEMI: ST-elevation myocardial infarction; TnT: troponin T; eGFR: estimated glomerular filtration rate; CHD: coronary heart disease; BMI: body mass index; BNP: B-type natriuretic peptide; and hsCRP: high-sensitivity C-reactive protein.

**Table 2:** Baseline characteristics of male patients arranged according to quartiles of 25(OH)D.

Characteristics *n* (%)	Quartiles of 25(OH)D				*P*-value
	Q1	Q2	Q3	Q4	
25(OH)D (nM)*	34.3 ± 5.4	48.7 ± 3.1	59.3 ± 2.9	75.3 ± 12.5	0.000
Age, years*	65.3 ± 13.0	59.5 ± 13.3	57.1 ± 12.4	56.9 ± 13.0	0.000
Smoking status, *n* (%)					0.891
Current smoker, *n* (%)	53 (37.3)	46 (32.2)	48 (33.1)	43 (29.7)	
Past smoker, *n* (%)	55 (38.7)	62 (43.4)	63 (43.4)	63 (43.4)	
Never smoked, *n* (%)	34 (23.9)	35 (24.5)	34 (23.4)	39 (26.9)	
Angina pectoris, *n* (%)	33 (22.6)	41 (27.9)	39 (26.5)	30 (20.4)	0.414
CHF, n (%)					
Killip class 2–4	32 (21.9)	16 (10.9)	19 (12.9)	27 (18.4)	0.040
CABG, n (%)	10 (7.0)	14 (9.7)	5 (3.4)	8 (5.5)	0.174
PCI, n (%)	22 (15.1)	15 (10.2)	17 (11.6)	16 (10.9)	0.581
Hypertension, n (%)	102 (69.9)	83 (56.5)	80 (54.4)	91 (61.9)	0.033
History of DM 1, n (%)	4 (2.8)	5 (3.4)	2 (1.4)	0 (0.0)	0.138
History of DM 2, n (%)	43 (30.3)	35 (24.1)	23 (15.9)	18 (12.4)	0.001
STEMI, n (%)	27 (18.9)	21 (14.7)	25 (17.2)	31 (21.2)	0.527
TnT release, n (%)	78 (53.4)	56 (38.1)	64 (43.5)	60 (40.8)	0.047
eGFR (*μ*mol L^−1^)*	80.6 ± 33.1	86.5 ± 26.3	85.2 ± 22.3	79.8 ± 25.1	0.089
Cholesterol^†^/statin, *n* (%)	28 (19.2)	21 (14.3)	22 (15.0)	21 (14.3)	0.606
Beta-blocker, *n* (%)	37 (25.9)	44 (30.3)	37 (25.7)	35 (24.1)	0.661
Known CHD, *n* (%)	56 (38.9)	63 (43.4)	50 (34.2)	49 (33.3)	0.255
BMI (kg/m^2^)*	28.1 ± 4.4	27.9 ± 3.8	29.1 ± 3.8	28.1 ± 3.6	0.057
BNP quartiles					0.014
Q1	34 (23.3)	36 (24.5)	44 (29.9)	33 (22.4)	
Q2	29 (19.9)	39 (26.5)	33 (22.4)	45 (30.6)	
Q3	29 (19.9)	36 (24.5)	42 (28.6)	40 (27.2)	
Q4	54 (37.0)	36 (24.5)	28 (19.0)	29 (19.7)	
hsCRP quartiles					0.009
Q1	20 (13.9)	38 (25.9)	42 (28.6)	43 (29.3)	
Q2	30 (20.8)	43 (29.3)	36 (24.5)	39 (26.5)	
Q3	46 (31.9)	38 (25.9)	31 (21.1)	33 (22.4)	
Q4	48 (33.3)	28 (19.0)	38 (25.9)	32 (21.8)	

*Mean ± SD

^†^Concentration > 250 mg/dL.

SD: standard deviation; 25(OH)D: 25-hydroxyvitamin D; CHF: congestive heart failure; MI: myocardial infarction; CABG: coronary artery bypass grafting; PCI: percutaneous coronary intervention; DM: diabetes mellitus; STEMI: ST-elevation myocardial infarction; TnT: troponin T; eGFR: estimated glomerular filtration rate; CHD: coronary heart disease; BMI: body mass index; BNP: B-type natriuretic peptide; and hsCRP: high-sensitivity C-reactive protein.

**Table 3:** Hazard ratios for 25(OH)D, BNP, and hsCRP in univariate and multivariate analyses in both gender.

	Females		Males	
	Univariate HR (95% CI)*	Multivariate HR (95% CI)*	Univariate HR (95% CI)*	Multivariate HR (95% CI)*
25(OH)D				
All-cause mortality	0.13 (0.06–0.32)	0.16 (0.06–0.42)	0.45 (0.26–0.79)	0.70 (0.36–1.34)
Cardiac death	0.04 (0.01–0.31)	0.08 (0.01–0.59)	0.30 (0.13–0.66)	0.38 (0.14–1.06)
Sudden cardiac death	0.06 (0.01–0.46)	0.15 (0.02–1.20)	0.20 (0.07–0.60)	0.29 (0.08–1.03)
BNP				
All-cause mortality	9.02 (3.84–21.19)	4.58 (1.56–13.45)	2.86 (1.60–5.09)	1.79 (0.91–3.54)
Cardiac death	12.77 (2.99–54.48)	2.64 (0.57–12.18)	6.04 (2.33–15.68)	4.22 (1.42–12.58)
Sudden cardiac death	7.22 (1.62–32.31)	1.90 (0.31–11.73)	5.87 (1.71–20.16)	4.55 (1.01–20.44)
hsCRP				
All-cause mortality	2.43 (1.31–4.48)	1.60 (0.80–3.17)	2.30 (1.31–4.03)	2.10 (1.12–3.94)
Cardiac death	1.97 (0.83–4.71)	0.83 (1.02–1.11)	1.32 (0.65–2.69)	1.08 (0.47–2.48)
Sudden cardiac death	3.07 (0.98–9.65)	1.13 (0.32–4.06)	1.62 (0.66–3.95)	1.40 (0.52–3.81)

25(OH)D: 25-hydroxyvitamin D; BNP: B-type natriuretic peptide; hsCRP: high-sensitivity C-reactive protein; HR: hazard ratio; CI: confidence interval.

*Highest quartile (Q4) versus lowest quartile (Q1). 25(OH)D, BNP, and hsCRP levels are highest in Q4.
